# Effects of Elevated Carbon Dioxide and Chronic Warming on Nitrogen (N)-Uptake Rate, -Assimilation, and -Concentration of Wheat

**DOI:** 10.3390/plants9121689

**Published:** 2020-12-01

**Authors:** Dileepa M. Jayawardena, Scott A. Heckathorn, Jennifer K. Boldt

**Affiliations:** 1 Department of Environmental Sciences, University of Toledo, Toledo, OH 43606, USA; dileepa.jayawardena@rockets.utoledo.edu; 2 Agricultural Research Service, United States Department of Agriculture, Toledo, OH 43606, USA; jennifer.boldt@usda.gov

**Keywords:** climate change, elevated CO_2_, warming, heat stress, nitrogen metabolism, N uptake, N assimilation, ^15^N tracer, *Triticum*, wheat

## Abstract

The concentration of nitrogen (N) in vegetative tissues is largely dependent on the balance among growth, root N uptake, and N assimilation. Elevated CO_2_ (eCO_2_) plus warming is likely to affect the vegetative-tissue N and protein concentration of wheat by altering N metabolism, but this is poorly understood. To investigate this, spring wheat (*Triticum aestivum*) was grown for three weeks at two levels of CO_2_ (400 or 700 ppm) and two temperature regimes (26/21 or 31/26 °C, day/night). Plant dry mass, plant %N, protein concentrations, NO_3_^−^ and NH_4_^+^ root uptake rates (using ^15^NO_3_ or ^15^NH_4_), and whole-plant N- and NO_3_^-^-assimilation were measured. Plant growth, %N, protein concentration, and root N-uptake rate were each significantly affected only by CO_2_, while N- and NO_3_^−^-assimilation were significantly affected only by temperature. However, plants grown at eCO_2_ plus warming had the lowest concentrations of N and protein. These results suggest that one strategy breeding programs can implement to minimize the negative effects of eCO_2_ and warming on wheat tissue N would be to target the maintenance of root N uptake rate at eCO_2_ and N assimilation at higher growth temperatures.

## 1. Introduction

Wheat (*Triticum aestivum*) ranks third among field crops globally and in the United States (U.S.) in terms of production, behind corn (*Zea mays*) and rice (*Oryza sativa*) globally or corn and soybean (*Glycine max*) nationally [1,2]. Wheat grain protein content is a major determinant of baking quality and it largely depends on the nitrogen (N) concentration during the vegetative stage of growth because the grains receive most of their N from vegetative tissues via remobilization [3,4]. Though tissue N can be enhanced by improved N fertilization, the drawbacks of using more N fertilizer to boost N content include higher costs of production and environmental problems caused by excess application of N fertilizers. The concentration of N in vegetative tissues is largely dependent on the balance among growth, root N uptake, and N assimilation [5,6]. Climate change will impact plant growth and N metabolism, but these impacts have mostly been studied to date by examining the effects of individual climate-change factors (especially eCO_2_, higher temperatures, and drought). However, since these main climate-change factors will change concomitantly, discerning the interactive effects of these factors will be necessary to understand how climate change will impact plant N metabolism.

Many studies have investigated the individual effects of eCO_2_ or warming on plant N relations, but data pertaining to the interactive effects of these two variables on N relations are scarce. To illustrate, the effects of eCO_2_ plus warming on tissue N and protein concentrations of wheat and other species are very limited. Studies have shown that eCO_2_ plus warming can reduce leaf N concentration of wheat [7,8]. Most past studies that have investigated the interactive effects of eCO_2_ and warming on tissue N concentration of species other than wheat found a non-significant effect on root N concentration [9,10,11,12,13,14], but a trend towards decreasing shoot or leaf N concentration [9,15,16,17]. However, there are reports that show that eCO_2_ plus warming can either increase or decrease root N concentration [11,13,17], while effects on the shoot or leaf N concentration can be positive [8,10] or neutral [10,12,18,19]. Collectively, these studies indicate that the response of plant N concentration to eCO_2_ plus warming can be variable, likely due in part to differences in experimental protocols and plant species.

Effects of eCO_2_ plus warming on the vegetative tissue protein concentration of wheat has scarcely been studied. A study conducted by Jauregui et al. (2015) [7] found a negative effect of eCO_2_ plus warming on flag-leaf total-soluble protein concentrations of wheat. None of the previous studies investigated eCO_2_ plus warming effects on whole-plant protein concentration of wheat. Previously, Jayawardena et al. (2017) [10] examined the effects of eCO_2_ plus warming on the total root protein concentration of tomato (*Solanum lycopersicum*) provided either NO_3_^−^ or NH_4_^+^ as the sole N source, and they noted significant decreases of root protein concentration in both sets of plants in response to eCO_2_ plus warming.

In addition, the effects of eCO_2_ plus warming on plant N uptake have rarely been studied. The two studies that examined this in wheat measured N uptake as the amount of N in above-ground organs (g m^−2^), without taking root N into account, and both studies found no interactive effect of eCO_2_ and temperature on wheat N uptake [8,20]. In contrast, research from other species suggests an inconsistent effect of N-uptake rate in response to eCO_2_ plus warming. For example, a growth chamber study showed that eCO_2_ (700 vs. 400 ppm) plus warming (38 vs. 28 °C) reduced N-uptake rate of the C_3_ species *Abutilon theophrasti*, but they saw varying N-uptake rates with the C_4_ species *Amaranthus retroflexus* at different plant growth stages [21]. Meanwhile, no significant effect of eCO_2_ (510 ppm vs. ambient) plus warming on both NO_3_^−^ and NH_4_^+^-uptake rates of *Calluna vulgaris* and *Deschampsia flexuosa* was observed [22]. Using sequential harvesting, we previously examined the N-uptake rate of *S. lycopersicum* in response to CO_2_ (700 vs. 400 ppm) plus warming (37 vs. 30 °C or 38 vs. 33 °C) and discovered that eCO_2_ plus warming inhibited N-uptake rate [10].

As with N-uptake rate, N assimilation in response to eCO_2_ plus warming has rarely been studied, and collective results from the few studies suggest a tendency for N assimilation to decrease in response to eCO_2_ plus warming. Based on a gene-expression analysis in *Triticum durum*, eCO_2_ (700 vs. 370 ppm) plus warming (ambient + 4 °C) may inhibit N assimilation [23]. Similarly, a greenhouse experiment also reported that eCO_2_ (700 vs. 400 ppm) plus warming (ambient + 4 °C) inhibited N assimilation in flag leaves of *T. durum* [7]. In a previous study, based on root %N and protein data of *S. lycopersicum*, we suggested that eCO_2_ (700 vs. 400 ppm) plus warming (37 vs. 30 °C) inhibited root N assimilation [10].

The above-mentioned studies demonstrate that the effects of eCO_2_ plus warming on plant N metabolism are poorly understood, especially in wheat. A better understanding of wheat N metabolism in response to predicted future climate conditions is essential to improve the N-use efficiency of wheat. Therefore, the objective of this study was to determine the individual and interactive effects of eCO_2_ and chronic warming on plant growth, root NO_3_^−^ and NH_4_^+^-uptake rates, whole-plant N- and NO_3_^−^-assimilation, and whole-plant protein concentration of wheat. Results of this study will help to plant breeders to develop new wheat cultivars better adapted to future climate conditions.

## 2. Results

**Figure 1:**
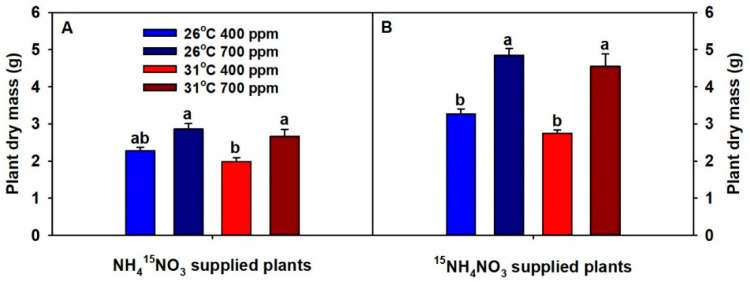
The effects of ambient (400 ppm) vs. elevated (700 ppm) CO_2_ and near-optimal (26 °C) vs. chronic warming (31 °C) daytime temperatures on total plant dry mass of *Triticum aestivum* cv. Glenn labeled for 3 days with 1 mM (A) NH_4_^15^NO_3_ or (B) ^15^NH_4_NO_3_ and grown for 21 or 23 days, respectively. Each bar represents mean (*n* = 4 or 5) + 1 standard error of mean (SEM). Within each panel, bars not sharing the same letters are significantly different (*p* < 0.05, Tukey’s test).

Across both temperatures, eCO_2_ significantly increased plant dry mass (except ^15^NO_3_^−^ supplied plants at 26 °C), while chronic warming insignificantly decreased it, and this was true for both NH_4_^15^NO_3_ and ^15^NH_4_NO_3_-supplied plants (i.e., in two independent sets of plants harvested two days apart) (Table S1, Figure 1).

**Figure 2:**
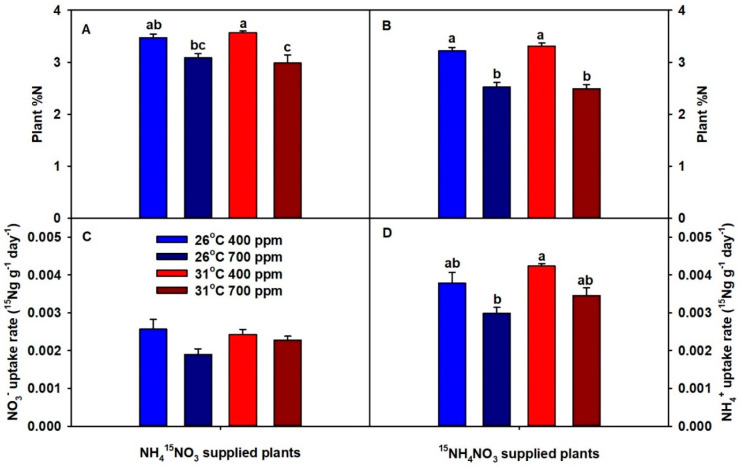
The effects of ambient (400 ppm) vs. elevated (700 ppm) CO_2_ and near-optimal (26 °C) vs. chronic warming (31 °C) daytime temperatures on (**A**) plant %N and (**C**) NO_3_^−^-uptake rate of NH_4_^15^NO_3_-supplied and (**B**) plant %N and (**D**) NH_4_^+^-uptake rate of ^15^NH_4_NO_3_-supplied *Triticum aestivum* cv. Glenn plants grown for 21 or 23 days, respectively. Each bar represents mean (*n* = 4 or 5) + 1 SEM. Within each panel, bars not sharing the same letters are significantly different (*p* < 0.05, Tukey’s test).

Across both temperatures, eCO_2_ significantly decreased plant %N (except ^15^NO_3_^−^ supplied plants at 26 °C), while chronic warming did not influence %N (Figure 2A,B). Notably, %N was lowest in plants grown at eCO_2_ plus warming. Across both temperatures, eCO_2_ decreased NO_3_^−^-uptake rate (more so at 26 °C) and NH_4_^+^-uptake rate (Figure 2C,D). In contrast, chronic warming did not affect NO_3_^−^-uptake rate but did marginally increase NH_4_^+^-uptake rates (Figure 2C,D). Among all treatment combinations, NH_4_^+^-uptake rates were consistently greater than NO_3_^−^-uptake rates.

**Figure 3:**
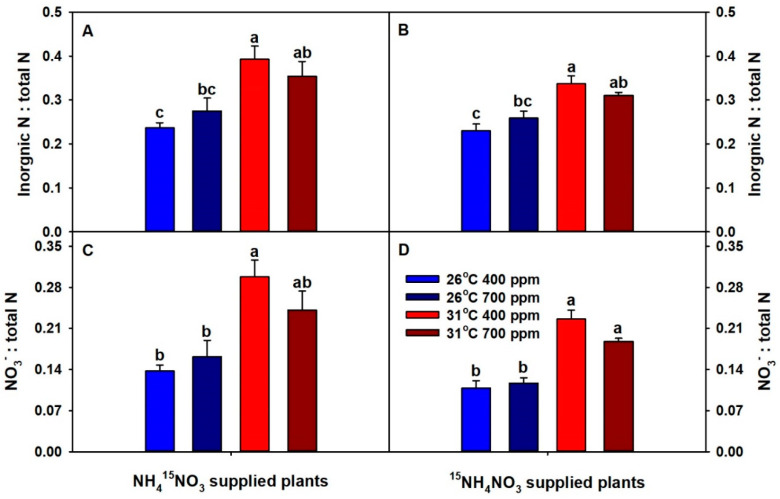
The effects of ambient (400 ppm) vs. elevated (700 ppm) CO_2_ and near-optimal (26 °C) vs. chronic warming (31 °C) daytime temperatures on total-plant (**A,B**) inorganic N:total N and (**C,D**) NO_3_^−^: total N ratios of either NH_4_^15^NO_3_ or ^15^NH_4_NO_3_ supplied *Triticum aestivum* cv. Glenn plants grown for 21 or 23 days, respectively. Each bar represents mean (*n* = 4 or 5) + 1 SEM. Within each panel, bars not sharing the same letters are significantly different (*p* < 0.05, Tukey’s test).

The ratios of total-plant inorganic N:total N and total-plant NO_3_^−^:total N were significantly affected only by temperature (Table S1). Elevated CO_2_ non-significantly increased the inorganic N:total N and NO_3_^−^:total N ratios at 26 °C, while decreasing the ratios at 31 °C (Figure 3). In contrast, chronic warming significantly or non-significantly increased the inorganic N:total N and NO_3_^−^:total N ratios across CO_2_ levels.

**Figure 4:**
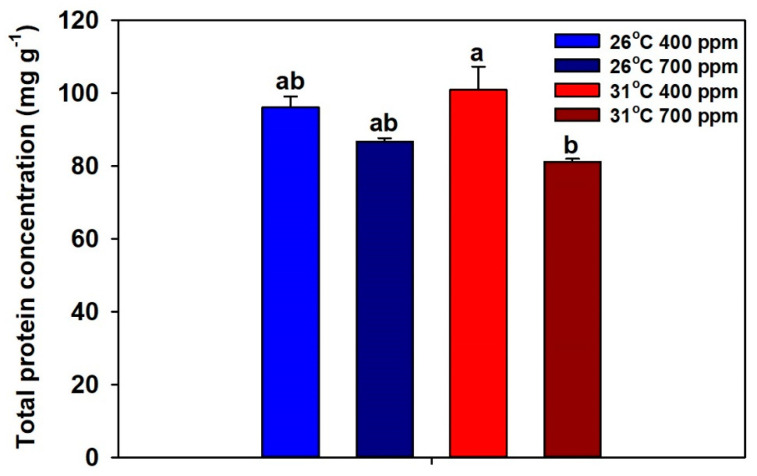
The effects of ambient (400 ppm) vs. elevated (700 ppm) CO_2_ and near-optimal (26 °C) vs. chronic warming (31 °C) daytime temperatures on whole-plant total-protein concentration of *Triticum aestivum* cv. Glenn labeled with 1 mM NH_4_^15^NO_3_. Each bar represents mean (*n* = 4) + 1 SEM. Within each panel, bars not sharing the same letters are significantly different (*p* < 0.05, Tukey’s test).

The whole-plant total-protein concentration was significantly reduced only by CO_2_ (Table S1, Figure 4).

## 3. Discussion

In the present study, eCO_2_ enhanced the growth of wheat irrespective of the temperature. In contrast, though statistically not significant, warming caused a slight decrease in plant growth at each CO_2_ level. Previously, we noticed severe inhibition of tomato growth caused by the combination of eCO_2_ and warming [10,24], which was partly due to a dramatic increase in leaf angle, and thus decrease in photosynthesis, compared to eCO_2_ or warming alone [24]. This growth response was not observed in wheat. These results indicate that interactive effects of CO_2_ enrichment and warming will be species-specific, and not necessarily additive as observed in tomato.

Typically, plants grown under eCO_2_ have lower tissue N concentrations due to increased photosynthetic assimilation of C and reduced N uptake caused by various reasons, such as the closure of stomates, down-regulation of Ribulose-1,5-bisphosphate carboxylase/oxygenase (RUBISCO), and increased lateral roots-to-primary roots ratio [6]. Similarly, in this study, wheat plants grown under eCO_2_ had lower plant %N regardless of the temperature treatment; moreover, plants grown at eCO_2_ plus warming had the lowest plant %N. Elevated CO_2_-driven growth stimulation and decreased %N indicate that plants cannot increase their N-uptake rate to keep pace with enhanced plant growth under eCO_2_. In fact, both NO_3_^−^ and NH_4_^+^-uptake rates decreased slightly with eCO_2_ at 26 °C; however, both NO_3_^−^ and NH_4_^+^-uptake rates were unaffected by eCO_2_ at 31 °C. These results indicate that the plants grown at eCO_2_ plus warming were able to maintain N-uptake rate per unit root, yet tissue N concentration in these plants still decreased, which could be due to growth dilution. Meanwhile, chronic warming at ambient CO_2_ (aCO_2_) caused a slight increase in plant %N, and it was correlated with a marginal increase in NH_4_^+^-uptake rate. Although the NO_3_^−^-uptake rate is favored over NH_4_^+^-uptake rate by rising soil or root temperatures in some species [25], in the present study, wheat NO_3_^−^-uptake rate was unaffected by warming treatment.

In this study, the ratios of inorganic N:total N and NO_3_^-^:total N were used as indices for whole-plant N- and NO_3_^−^-assimilation, respectively. Chronic warming increased both inorganic N:total N and NO_3_^−^:total N ratios, irrespective of the CO_2_ treatment, suggesting inhibition of both N and NO_3_^−^ assimilation by warming. In addition, the magnitude of warming-driven inhibition was partially offset by eCO_2_. Based on these results, we expected plants grown at eCO_2_ plus 31 °C to have low protein levels, and plants grown at aCO_2_ plus 31 °C to have the lowest protein levels. However, the protein data were in partial disagreement with these predictions. As expected from inorganic N:total N and NO_3_^−^:total N ratios, plants grown at eCO_2_ plus warming had the lowest protein concentration, which suggests that the low protein levels were due to the inhibition of N and NO_3_^−^ assimilation by eCO_2_ plus warming. Previously, Jauregui et al. (2015) [7] reported inhibition of leaf N assimilation in *T. durum* grown at eCO_2_ plus warming, and they proposed that the inhibition could be due to decreased leaf photorespiration and dark respiration. However, in this study, plants grown at aCO_2_ plus warming had the highest protein concentration, as well as the highest ratios of inorganic N:total N and NO_3_^−^:total N among all treatment combinations, indicating an accumulation of inorganic N forms. Though statistically not significant, eCO_2_ tended to decrease N- and NO_3_^−^-assimilation at 26 °C, and, as a result, plants grown at eCO_2_ plus 26 °C tended to have low protein concentrations.

In summary, eCO_2_ reduced wheat tissue N and protein concentrations regardless of the temperature treatment, but the magnitude of decrease tended to be greater when combined with warming. This decrease was not due to the decreased N-uptake rate, since both NO_3_^−^ and NH_4_^+^-uptake rates were not lowest in the eCO_2_ plus warming treatment. As ratios of inorganic N:total N and NO_3_^−^:total N suggest, low protein levels at eCO_2_ plus warming were likely due to the inhibition of N assimilation. To date, eCO_2_ plus warming effects on yield components of wheat have been studied in some detail [8,20,26,27]. In the present study, we investigated the N relations of wheat when plants were in the vegetative stage. Wheat plants reach their maximum N content when they are nearing anthesis [28], and about 50–90% of the final grain N is accumulated before anthesis and later remobilized from vegetative tissues to grains [3,4,29]. Therefore, the changes in N relations at the vegetative stage that we observed are likely to have a direct impact on determining final wheat grain quality. Together, these results indicate that crop improvement strategies, such as transgenic approaches, genetic engineering, and traditional plant breeding, might focus on developing wheat cultivars which can maintain root N uptake rate at eCO_2_ and N assimilation at higher growth temperatures in order to enhance the nutritional quality of wheat in a warming world enriched with CO_2_.

## 4. Materials and Methods 

### 4.1. Plant Material, Growth Conditions, and Treatments

Wheat (*T. aestivum* L. cv. Glenn), which is a cool-season C_3_ grass, was used as the model species. It is a hard-red spring wheat cultivar developed by North Dakota State University (NDSU, Fargo, ND, USA) for high yields, high protein, and scab resistance, and it was released by the North Dakota Agricultural Experiment Station in 2005 [30].

Seeds were sown in trays filled with calcined clay in a greenhouse and watered daily. Air temperature in the greenhouse fluctuated between 25–30 °C. When photosynthetically active radiation (PAR) dropped below 300 µmol m^−2^ s^−1^, 250-W high-pressure sodium (GE Lighting Inc., Cleveland, OH, USA) and 400-W metal-halide (Osram Sylvania Products Inc., Manchester, NH, USA) lamps provided supplementary lighting to maintain a 15-h photoperiod. When seedlings were 12-cm tall, 44 seedlings having two leaves were transplanted into 3.1 L cylindrical pots (10-cm diameter × 40-cm length PVC pipes; one plant per pot) containing a mixture of calcined clay and perlite in a 3:1 (*v:v*) ratio and supported by mesh at the bottom of the pot.

Pots were transferred to four growth chambers (model E36HO, Percival Scientific Inc., Perry, IA, USA), with 11 pots per chamber. A 2 × 2 factorial experimental design was used, with CO_2_ levels of ambient (400 ppm) vs. elevated (700 ppm) and temperatures of near-optimal (26/21 °C; day/night) vs. chronic warming (31/26 °C; day/night). According to the low and intermediate CO_2_ emission scenarios, atmospheric CO_2_ is likely to be between 450–1000 ppm by 2100 [31]. Therefore, we chose 700 ppm as our eCO_2_ treatment, which is an intermediate value between today’s CO_2_ level and the upper limit of the intermediate CO_2_ emission scenario. Near-optimal and supra-optimal temperatures were chosen based on preliminary experiments. Plants were acclimated to the new chamber environment for four days at 600 µmol m^−2^ s^−1^ PAR (supplied by 55-W Osram Dulux luminous lamps; Osram GmbH, Augsburg, Germany) with a 14-h (0600-2000 HR) photoperiod, 26/21 °C (day/night near-optimum growth temperatures), ambient CO_2_ (400 ppm), and ambient humidity. During this period, 500 mL of quarter-strength nutrient solution was added to each pot twice (nutrient concentrations of the full-strength solution: 2 mM MgSO_4_, 1 mM KH_2_PO_4_, 1 mM K_2_HPO_4_, 2 mM CaCl_2_, 71 µM Fe-DTPA, 10 µM MnCl_2_, 50 µM H_3_BO_3_, 6 µM CuSO_4_, 6 µM ZnSO_4_, 1 µM Na_2_MoO_4_, 1 mM NH_4_NO_3_; pH = 6.0). When plants were free from transplant-stress, the temperature of the high-temperature-treatment chambers was gradually increased from 26 °C to 31 °C over three days to avoid potential heat-shock. Once high-temperature-treatment chambers reached 31 °C, CO_2_ treatments were started (day 0). Plants were fertilized with 500 mL of full-strength complete nutrient solution every other day. They were rotated within chambers every 4–5 days to avoid potential position effects and switched between chambers every 7–8 days to avoid potential chamber effects.

The nutrient solution with stable isotopes was made by adding either NH_4_^15^NO_3_ or ^15^NH_4_NO_3_ with an isotopic purity of 98 atom% ^15^N (Sigma-Aldrich Inc., St. Louis, MO, USA). Carbenicillin (an antibiotic) was mixed (20 mg L^−1^) with the labeled nutrient solution to avoid external nitrification, de-nitrification, and N immobilization by microbes (based on preliminary experiments and Smart et al., 1995 [32]). On day 18, a subset of plants (*n* = 4) from each chamber was flushed with 1950 mL (pore volume of the 3.1 L pot) of full-strength complete nutrient solution containing 1 mM NH_4_^15^NO_3_. On day 19, another subset of plants (*n* = 2) from each chamber was flushed with 1950 mL of un-labeled complete nutrient solution (controls to determine ^15^N background levels). On day 20, the rest of the plants (*n* = 5) were flushed with 1950 mL of the nutrient solution containing 1 mM ^15^NH_4_NO_3_. Each set of plants was harvested three days after labeling (based on preliminary experiments, Figure S1). When harvesting, plants were at their stem elongation stage before booting.

### 4.2. Plant N and Protein Analysis

Harvested plants were split into shoots and roots. Roots were cleaned with tap water. Each root and shoot system was longitudinally divided into two halves using a scissor and weighed separately to the nearest 0.001 gram. One half from each was flash-frozen in liquid N_2_ and stored at −80 °C to be used for protein and NH_4_^+^ quantification (fresh tissue). The other half from each plant was oven-dried (model 760F, Fisher Scientific, Waltham, MA, USA) at 70 ± 0.1 °C for 72 h and then weighed (the dry: fresh mass ratio of this half of the plant was used to estimate dry mass for the other half).

Dried shoot and root samples were ground and homogenized into a fine powder using a coffee grinder (model 11160-3, Bodum, Triengen, Switzerland) and a subset of samples (ca. 0.01 g) were analyzed for %C and %N via the combustion-MS technique [33], using one technical replicate for each biological replicate. Another subset of samples (one technical replicate for each biological replicate) was sent to the University of California Stable Isotope Facility to analyze ^15^N (atom %) in solid samples using an elemental analyzer interfaced to a continuous flow isotope ratio mass spectrometer. Nitrate and NH_4_^+^ -uptake rates were calculated as the total amount of ^15^N taken up by plants treated with ^15^NO_3_^−^ or ^15^NH_4_^+^ per g of dry root per day (using root mass at the final harvest). Background ^15^N was subtracted using ^15^N (atom %) of unlabeled plants. Tissue NO_3_^−^ was quantified according to the method described in Cataldo et al. (1975) [34] using two technical replicates for each biological replicate. Briefly, 50 mg of ground dried tissue was suspended in 5 mL of de-ionized water. After incubation at 45 °C for 1 h and centrifugation (model 5810R, Eppendorf, Framingham, MA, USA) at 10,000 rpm for 5 min at room temperature, the supernatant was recovered. Then, 0.2 mL of recovered supernatant was reacted with 0.8 mL of 5% (*w/v*) salicylic acid-sulfuric solution and 19 mL of 2N NaOH. Nitrate was quantified by measuring absorbance (model UV-1650-PC, Shimadzu, Columbia, MD, USA) at 410 nm using KNO_3_ standards with concentration ranging from 10–60 µg mL^−1^. When quantifying NH_4_^+^, 500 mg of fresh tissue was ground into a fine powder using liquid N_2_. Then, NH_4_^+^ was extracted into 30 mL of 0.001M acidic CaSO_4_ solution (pH = 3) and quantified using an NH_4_^+^ ion-selective electrode (Hach company, Loveland, CO, USA) using two technical replicates for each biological replicate. The total plant inorganic N content was estimated as the sum of total-NO_3_^−^ and -NH_4_^+^ contents with which the ratios of total inorganic N:total N and total NO_3_^−^:total N were calculated.

Shoot and root proteins were extracted as described in Jayawardena et al. (2017) [10]. Briefly, 1 g of fresh tissue was ground to a fine powder with liquid N_2_ and then with 4 mL of an extraction buffer [0.2 M Tris-HCl, pH = 8.0; 5 mM ethylenediaminetetraacetic acid (EDTA), pH = 7.5–8.0; 0.7 M sucrose; 1% sodium dodecyl sulfate (SDS); 1 mM phenylmethylsulfonyl fluoride; 1 mM leupeptin; and 2% β-mercaptoethanol]. Protein was extracted into a phenol phase by adding an equal volume of buffer-saturated phenol (pH = 6.6–8.0) following centrifugation (10,000 rpm for 10 min at 4 °C). Extracted protein was pelleted down by centrifugation (10,000 rpm for 10 min at 4 °C) after adding five volumes of 0.1 M ammonium acetate in methanol. The protein pellet was washed several times with ammonium acetate and 100% acetone. Then, the protein pellet was air-dried within less than a min under room temperature. Each root and shoot protein pellet was added to 1.6 mL and 3 mL of a re-solubilizing buffer (62.5 mM Tris-HCl, pH = 6.8; 0.5 SDS; 20% glycerol), respectively, to dissolve them. Protein concentration was determined by measuring absorbance (model UV-1650-PC, Shimadzu) at 750 nm using a colorimetric assay (DC protein assay; Bio-Rad Laboratories Inc., Hercules, CA, USA) and bovine serum albumin (BSA) as the protein standard. Two and three technical replicates were used for each unknown and standard sample, respectively.

### 4.3. Statistical analysis

Data were analyzed using RStudio version 3.6.1 (RCore Team, Vienna, Austria). Statistical assumptions of independence, normality and equal variance were checked with residual vs. fitted, normal quantile-quantile (Q-Q) and spread-level (S-L) plots, respectively. If statistical assumptions were not met, data were transformed, and log transformation proved to be sufficient. Data were analyzed using two-way (two levels of CO_2_ × two levels of temperature) analysis of variance (ANOVA) with CO_2_ and temperature as fixed factors. Results were considered significant if *p* < 0.05. If ANOVA results were significant, Tukey’s post-hoc test was performed for multiple comparisons. Figures were generated using SigmaPlot version 14.0 (Systat Software, Inc., San Jose, CA, USA). Results presented in figures are untransformed means and SEM.

## Supplementary Materials

The following are available online at https://www.mdpi.com/2223-7747/9/12/1689/s1, Figure S1: Timeline of different steps in plant growth, treatments, and harvest, Table S1: *p*-values from ANOVA statistical analysis.

## References

[1] Wheat: Overview. https://www.ers.usda.gov/topics/crops/wheat/.

[2] Shewry P.R., Hey S.J. (2015). The contribution of wheat to human diet and health. Food Energy Secur..

[3] Barneix A.J. (2007). Physiology and biochemistry of source-regulated protein accumulation in the wheat grain. J. Plant Physiol..

[4] Gregersen P.L., Holm P.B., Krupinska K. (2008). Leaf senescence and nutrient remobilisation in barley and wheat. Plant Biol..

[5] Bloom A.J., Burger M., Asensio J.S.R., Cousins A.B. (2010). Carbon dioxide enrichment inhibits nitrate assimilation in wheat and *Arabidopsis*. Science.

[6] Taub D.R., Wang X. (2008). Why are nitrogen concentrations in plant tissues lower under elevated CO_2_? A critical examination of the hypotheses. J. Integr. Plant Biol..

[7] Jauregui I., Aroca R., Garnica M., Zamarreño Á.M., García-Mina J.M., Serret M.D., Parry M., Irigoyen J.J., Aranjuelo I. (2015). Nitrogen assimilation and transpiration: Key processes conditioning responsiveness of wheat to elevated [CO_2_] and temperature. Physiol. Plant..

[8] Cai C., Yin X., He S., Jiang W., Si C., Struik P.C., Luo W., Li G., Xie Y., Xion Y. (2016). Responses of wheat and rice to factorial combinations of ambient and elevated CO_2_ and temperature in FACE experiments. Glob. Chang. Biol..

[9] Dijkstra F.A., Blumenthal D., Morgan J.A., Pendall E., Carrillo Y., Follett R.F. (2010). Contrasting effects of elevated CO_2_ and warming on nitrogen cycling in a semiarid grassland. New Phytol..

[10] Jayawardena D.M., Heckathorn S.A., Bista D.R., Mishra S., Boldt J.K., Krause C.R. (2017). Elevated CO_2_ plus chronic warming reduce nitrogen uptake and levels or activities of nitrogen-uptake and -assimilatory proteins in tomato roots. Physiol. Plant..

[11] King J.S., Thomas R.B., Strain B.R. (1997). Morphology and tissue quality of seedling root systems of *Pinus taeda* and *Pinus ponderosa* as affected by varying CO_2_, temperature, and nitrogen. Plant Soil.

[12] Li C., Zhu J.G., Sha L.N., Zhang J.S., Zeng Q., Liu G. (2017). Rice (*Oryza sativa* L.) growth and nitrogen distribution under elevated CO_2_ concentration and air temperature. Ecol. Res..

[13] Nelson L., Blumenthal D.M., Williams D.G., Pendall E. (2017). Digging into the roots of belowground carbon cycling following seven years of Prairie Heating and CO_2_ Enrichment (PHACE), Wyoming USA. Soil Biol. Biochem..

[14] Wan S., Norby R.J., Pregitzer K.S., Ledford J., O’Neill E.G. (2004). CO_2_ enrichment and warming of the atmosphere enhance both productivity and mortality of maple tree fine roots. New Phytol..

[15] Lilley J.M., Bolger T.P., Peoples M.B., Gifford R.M. (2001). Nutritive value and the nitrogen dynamics of *Trifolium subterraneum* and *Phalaris aquatica* under warmer, high CO_2_ conditions. New Phytol..

[16] Rosenthal D.M., Ruiz-Vera U.M., Siebers M.H., Gray S.B., Bernacchi C.J., Ort D.R. (2014). Biochemical acclimation, stomatal limitation and precipitation patterns underlie decreases in photosynthetic stimulation of soybean (*Glycine max*) at elevated [CO_2_] and temperatures under fully open air field conditions. Plant Sci..

[17] Salazar-Parra C., Aranjuelo I., Pascual I., Erice G., Sanz-Sáez Á., Aguirreolea J., Sánchez-Díaz M., Irigoyen J.J., Araus J.L., Morales F. (2015). Carbon balance, partitioning and photosynthetic acclimation in fruit-bearing grapevine (*Vitis vinifera* L. cv. Tempranillo) grown under simulated climate change (elevated CO_2_, elevated temperature and moderate drought) scenarios in temperature gradient greenhouses. J. Plant Physiol..

[18] Johnson S.N., Hartley S.E. (2018). Elevated carbon dioxide and warming impact silicon and phenolic-based defenses differently in native and exotic grasses. Glob. Chang. Biol..

[19] Ramalho J.C., Pais I.P., Leitão A.E., Guerra M., Reboredo F.H., Máguas C.M., Carvalho M.L., Scotti-Campos P., Ribeiro-Barros A.I., Lidon F.J.C. (2018). Can elevated air [CO_2_] conditions mitigate the predicted warming impact on the quality of coffee bean?. Front. Plant Sci..

[20] Wang J., Hasegawa T., Li L., Lam S.K., Zhang X., Liu X., Pan G. (2019). Changes in grain protein and amino acids composition of wheat and rice under short-term increased [CO_2_] and temperature of canopy air in a paddy from East China. New Phytol..

[21] Coleman J.S., Bazzaz F.A. (1992). Effects of CO_2_ and temperature on growth and resource use of co-occurring C_3_ and C_4_ annuals. Ecology.

[22] Arndal M.F., Schmidt I.K., Kongstad J., Beier C., Michelsen A. (2014). Root growth and N dynamics in response to multi-year experimental warming, summer drought and elevated CO_2_ in a mixed heathland-grass ecosystem. Funct. Plant Biol..

[23] Vicente R., Pérez P., Martínez-Carrasco R., Usadel B., Kostadinova S., Morcuende R. (2015). Quantitative RT-PCR platform to measure transcript levels of C and N metabolism-related genes in durum wheat: Transcript profiles in elevated [CO_2_] and high temperature at different levels of N supply. Plant Cell Physiol..

[24] Jayawardena D.M., Heckathorn S.A., Bista D.R., Boldt J.K. (2019). Elevated carbon dioxide plus chronic warming causes dramatic increases in leaf angle in tomato, which correlates with reduced plant growth. Plant Cell Environ..

[25] Bassirirad H. (2000). Kinetics of nutrient uptake by roots: Responses to global change. New Phytol..

[26] Mitchell R.A.C., Mitchell V.J., Driscoll S.P., Franklin J., Lawlor D.W. (1993). Effects of increased CO_2_ concentration and temperature on growth and yield of winter wheat at two levels of nitrogen application. Plant Cell Environ..

[27] Zhang X., Shi Z., Jiang D., Högy P., Fangmeier A. (2019). Independent and combined effects of elevated CO_2_ and post-anthesis heat stress on protein quantity and quality in spring wheat grains. Food Chem..

[28] Wetselaar R., Farquhar G.D. (1980). Nitrogen losses from tops of plants. Adv. Agron..

[29] Masclaux-Daubresse C., Reisdorf-Cren M., Orsel M. (2008). Leaf nitrogen remobilisation for plant development and grain filling. Plant Biol..

[30] Glenn Hard Red Spring Wheat. http://ndsuresearchfoundation.org/glenn.

[31] IPCC (2014). Climate Change 2014: Synthesis Report. Contribution of Working Groups I, II, and III to the Fifth Assessment Report of the Intergovernmental Panel on Climate Change.

[32] Smart D.R., Ferro A., Ritchie K., Bugbee B.G. (1995). On the use of antibiotics to reduce rhizoplane microbial populations in root physiology and ecology investigations. Physiol. Plant..

[33] Mishra S., Heckathorn S., Frantz J., Yu F., Gray J. (2009). Effects of boron deficiency on geranium grown under different nonphotoinhibitory light levels. J. Am. Soc. Hortic. Sci..

[34] Cataldo D.A., Maroon M., Schrader L.E., Youngs V.L. (1975). Rapid colorimetric determination of nitrate in plant tissue by nitration of salicylic acid1. Commun. Soil Sci. Plant Anal..

