# Supplementary material for: Effects of Elevated Carbon Dioxide and Chronic Warming on Nitrogen (N)-Uptake Rate, -Assimilation, and -Concentration of Wheat
Source: Plants (Basel). 2020 Dec 1;9(12):1689. doi: 10.3390/plants9121689 (PMC7760685; doi:10.3390/plants9121689)
Supplement: Supplementary file 1 [file plants-09-01689-s001.pdf]

Supplementary Materials

Table S1. *p*-values from ANOVA statistical analysis.

	Response Variable	Temperature	CO_2	Temperature × CO_2
$NH_4{}^{15}NO_3$ supplied plants	Total plant dry mass (g)	0.0867	0.0007	0.5912
	Total plant %N	0.8836	0.0004	0.3142
	NO_3^- uptake rate (^{15}Ng g^{-1} root day^{-1})	0.3500	0.0308	0.1243
	Inorganic N: total N ratio	0.0011	0.9998	0.1851
	Total NO_3^- : total N	0.0007	0.5575	0.1487
	Total plant protein concentration (mg g^{-1})	0.6722	0.0024	0.2105
$^{15}NH_4NO_3$ supplied plants	Total plant dry mass (g)	0.0709	< 0.001	0.3513
	Total plant %N	0.8380	< 0.001	0.3980
	NH_4^+ uptake rate (^{15}Ng g^{-1} root day^{-1})	0.0508	0.0016	0.8616
	Inorganic N: total N ratio	< 0.001	0.9708	0.0832
	Total NO_3^- : total N	< 0.001	0.1874	0.0616

Note: *p*-values in blue are statistically significant.

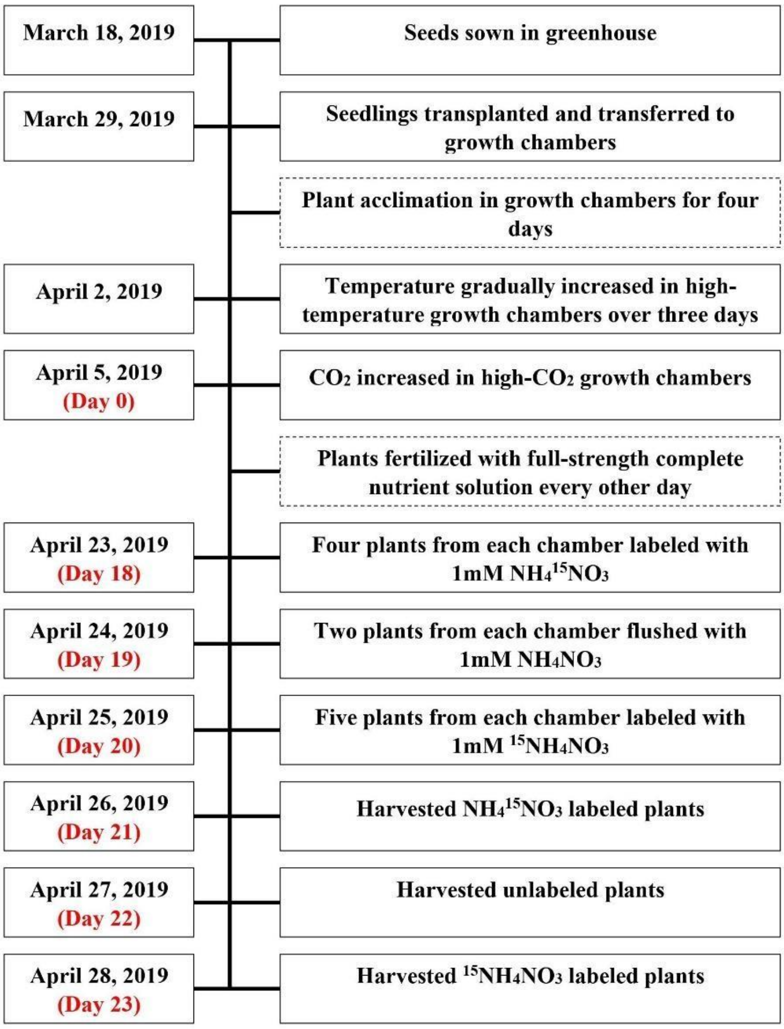

Figure 1. Timeline of different steps in plant growth, treatments, and harvest.
